# Supplementary material for: Complexome profiling of the Chlamydomonas psb28 mutant reveals TEF5 as an early PSII assembly factor
Source: Plant Cell. 2025 Mar 17;37(6):koaf055. doi: 10.1093/plcell/koaf055 (PMC12164590; doi:10.1093/plcell/koaf055)
Supplement: koaf055_Supplementary_Data [file koaf055_supplementary_data.zip › psb28_interactive.zip!/plots/intensity/qualityPlots/peptides/ELIP6_Cre17.g740950.t1.2.html]

### Cre17.g740950.t1.2

Trivial name: ELIP6  
SeqCoverage [median]: 25.60  
Mass: 30.16 kDa  
  
 

| Sequence | MissedCleavage | Mass [Da] | wtIntensity | muIntensity | Score |
| --- | --- | --- | --- | --- | --- |
| FLNVNGTEAAADAAASEMK | 0 | 1908.89 | 0.00 | 4370.43 | 47.09 |
| GKPDFASPGLDMAAIEATR | 1 | 1945.96 | 0.00 | 22709.06 | 86.54 |
| REPTMQDLYGVDDVTYAR | 1 | 2127.99 | 0.00 | 17355.43 | 116.29 |
| SSTATAVAEPPAAPAPK | 0 | 1564.81 | 0.00 | 7677.32 | 130.56 |
|  |  |  | 0.0 | 52112.2 |  |
